# Respirophasic Variations in the QRS Complex and Echocardiographic Equivalent in Pulmonary Embolism

**Published:** 2010-07-20

**Authors:** Eftychios Siniorakis, Spyridon Arvanitakis, Dimitris Barlagiannis, Antonis Samaras, Nikos Flessas, Constantinos Karidis

**Affiliations:** Department of Cardiology, Sotiria Chest Diseases Hospital, Athens, Greece.

**Keywords:** Pulmonary embolism, electrocardiogram, tricuspid annulus, tissue Doppler imaging

## Abstract

A respirophasic variation of the QRS complex was observed in a patient with bilateral pulmonary embolism and pulmonary hypertension. An analogous variation of the Sa systolic velocity of the tricuspid annular tissue Doppler imaging was also noticed. Both phaenomena persisted until the clinical and haemodynamic improvement of the patient. A mechanistic approach of the electric phaenomenon is proposed.

## Introduction

We enjoyed the article by K. Todd and co-authors, concerning the various patterns of electrocardiogram (ECG) in pulmonary embolism (PE) [1]. Another morphology is sometimes encountered, as in the PE we describe here. A respirophasic electric variation (EV) of the QRS complex was observed, accompanied by a tricuspid annular analogue, in the tissue Doppler imaging (TDI). A potential mechanistic and electric correlation is proposed.

## Introduction

**Figure 1:**
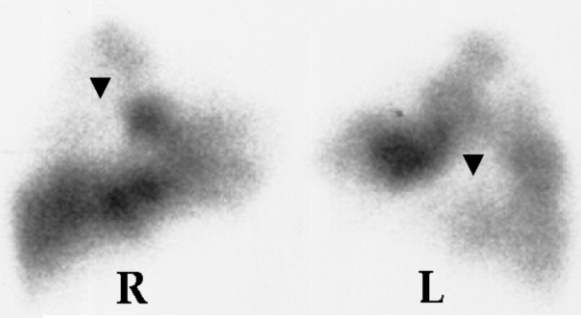
Lung scan shows multiple bilateral perfusion defects (arrows). R = right and L = left lung (lateral views)

A 62 year old male, with no previous cardiocirculatory or respiratory problems, was admitted because of bilateral PE confirmed by lung scintigraphy (Figure 1) and spiral computed tomography. On presentation, he was diaphoretic and dyspnoeic, with a systolic blood pressure of 80 mm Hg. Blood gas analysis showed hypoxaemia (PO2 = 60mmHg) and hypocapnia (PCO2 = 32 mm Hg). Venous ultrasonography revealed a deep right popliteal vein thrombosis. D-dimers were 20μg/ml (normal value ≤0.50μg/ml) and brain natriuretic peptide (BNP) 1620 pg/ml (normal value 0-100pg/ml). Troponin I was normal. We did not administer thrombolysis, due to a recent episode of gastric bleeding. The patient also refused any mechanical intervention in his pulmonary artery. Thus, inravenous unfractionated heparin was started.

**Figure 2:**
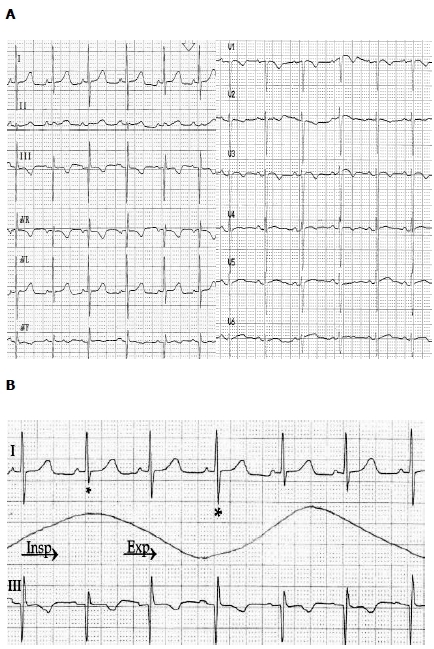
Panel A shows the admission electrocardiogram with an S1Q3T3 pattern, and varying amplitude of the QRS complex. In panel B, leads I and III are magnified, with simultaneous respirophasic monitoring. The size of the asterisk corresponds to the QRS amplitude. Insp=inspiration, Exp=expiration.

From the time of admission, an S1Q3T3 pattern on the ECG attracted our attention, because of a respirophasic EV of the QRS complex. This phenomenon was mainly apparent in leads I and III, which showed an inspiratory reduction of S, Q and R waves respectively, with restitution in expiration (Figure 2).

 A respirophasic echocardiogram (ECHO) was performed, paying attention to any potential cardiac positional or internal diameter changes. No such findings were detected. Left ventricle was normal in shape and performance. Pulmonary artery systolic pressure was calculated to 50 mm Hg. Right ventricle (RV) was not dilated but its middle and apical free wall were severely hypokinetic. No signs of abnormal ventricular interdependence were noticed. There were no pericardial or pleural effusions, and inferior vena cava had a slightly dilated diameter, with normal inspiratory behaviour.

**Figure 3:**
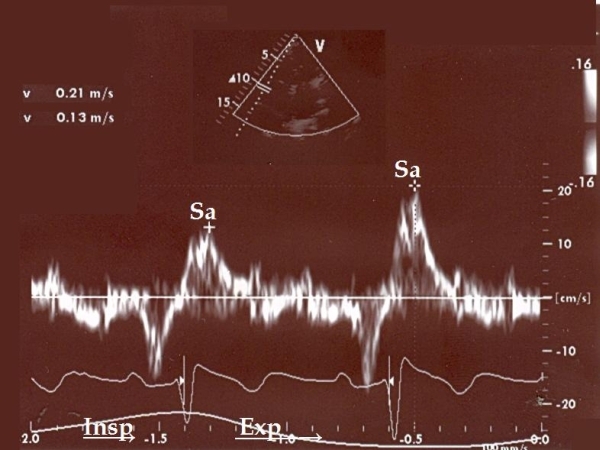
Respirophasic variations in the tricuspid annular systolic Sa velocity. An inspiratory decrease corresponding to 38% of the expiratory value is noticed.

When a TDI of the tricuspid annulus was reviewed, a respirophasic variation of the systolic Sa velocity appeared, in a parallel way with the EV (Figure 3). Thus, Sa significantly decreased in inspiration, and was restored in expiration. EV and TDI findings persisted until day 7, when the patient appeared clinically stable and weaning from supplemental nasal oxygen was achieved. D-dimers and BNP values on that day were 3 μg/ml and 280 pg/ml respectively. The patient was discharged on day 10, on oral anticoagulants. Discharge ECHO showed a pulmonary artery systolic pressure reduced to 35 mm Hg, while the RV free wall hypokinesis persisted, although somewhat improved.

## Discussion

EV of the QRS complex in the setting of PE is a rare phaenomenon with rather unclarified aetiology. Another case was published in 1994, where the EV acquired the form of electric alternans, without obvious respirophasic correlations. The authors of that case, attributed the EV to ischaemia of the RV when stressed by the PE [2].

Recently, the tricuspid annular TDI came to shed a light in the study of patients with PE [3,4]. However, no respirophasic measurements have been published until now. In our case, an unexpected respirophasic behaviour of the Sa velocity appeared in the tricuspid annular TDI. This phenomenon was in-phase with EV, persisted as long as the EV, and they both disappeared simultaneously. The parallel presence of these ECG and TDI manifestations warrants the search for a common aetiology. If we admit that S1Q3T3 ECG pattern represents acute cor pulmonale, then, stressing and secondary ischaemia of RV are awaited, especially if pulmonary hypertension ensues, as in this case [5].

Inspiration provokes an increased venous return, which further compromises the functional status of the RV. This could result in an inspiratory reduction of the tricuspid annular Sa velocity, which is known to correlate with the RV performance, particularly the RV ejection fraction [6]. This functional inspiratory impairment, could also explain the QRS voltage reduction in leads related to the acute stressing of RV, as the leads I and III are [7,8]. We do not know if the theory we adopt here to interpret the synchronous electric and ECHO variations is the only available. PE in this case, was rather severe, as shown by the pulmonary hypertension, BNP values (exclusively due to RV stressing as far as the left ventricle was completely preserved), and persisting RV hypokinesis. Therefore electric and Sa variations, could be viewed as the two sides of the same phenomenon.

## Conclusion

Respirophasic EV is another potential ECG pattern in cases of PE. This finding and its ECHO equivalent, from the RV free wall TDI, should be viewed as markers of considerable haemodynamic compromise. We further thank K. Todd and coauthors, for giving us the opportunity to report an intriguing ECG manifestation of PE.
